# Double resolvability parameters of fosmidomycin anti-malaria drug and exchange property

**DOI:** 10.1016/j.heliyon.2024.e33211

**Published:** 2024-06-21

**Authors:** Rashad Ismail, Sikander Ali, Muhammad Azeem, Manzoor Ahmad Zahid

**Affiliations:** a Department of Mathematics, Faculty of Science and Arts, Mahayl Assir, King Khalid University, Abha, Saudi Arabia; b Department of Mathematics, COMSATS University Islamabad, Sahiwal campus, Pakistan; c Department of Mathematics, Riphah International University Lahore, Pakistan

**Keywords:** Resolving set, Metric dimension, Edge metric dimension, Mixed metric dimension, Fosmidomycin, Exchange property

## Abstract

The practical and theoretical significance of the resolvability parameter makes it an important factor, particularly in the context of network analysis. Its significance is seen in various applications and consequences: Network security, efficient routing, social network analysis, facility location, and site selection. This article finds the double resolvability parameters of the fosmidomycin anti-malaria drug. Resolvability parameters like double metric, double edge metric, and double mixed metric dimensions of fosmidomycin anti-malaria drug also hold exchange properties in the molecular graph of fosmidomycin. We convert the molecular structures of fosmidomycin into molecular graphs and then find some resolvability parameters.

## 1 Introduction

Chemical graph theory serves as a valuable framework for analyzing chemical arrangements and representing molecular structures mathematically. This approach aids in describing the structural attributes of various substances, including crystals, processes, clusters, molecules, and polymers. By leveraging chemical graph theory, it becomes possible to examine chemical structures from multiple perspectives in emerging mathematical chemistry. This discipline combines discoveries and developments into mathematical models that represent chemical processes and the application of mathematical ideas and techniques to real-world chemistry [1]. Chemical graph theory is constructive when building and simplifying complex chemical structures that are difficult to understand in their original, unabstracted forms.

The drug fosmidomycin, which is used to treat and prevent malaria, is precise and educational. Millions of people worldwide suffer from malaria, a condition that these drugs are essential in fighting. To guarantee correct dosage and to keep an eye out for any possible adverse effects, those using these medications must do so under the supervision of medical specialists. Choosing the right antibiotic for treating malaria also requires taking into account drug resistance patterns and recommendations specific to the area.

Slater first proposed the idea of a locating set for a graph in 1975 [2]. Harary later introduced the same idea in 1976 under the name metric dimension of a graph [3]. The ideas of resolving sets and metric dimensions were previously described by Blumenthal in his monograph, which discussed metric spaces and applications of distance geometry in a broader context [4].

Several scholars have been attracted by the idea of metric dimension. For instance, Hernando et al. explored this idea by investigating the fault-tolerant resolution set of tree graphs, demonstrating that $P_{n}$ for n≥2 in the case of path graphs with n>2 vertices [5], [6]. Voronov estimated the FTMD of the king's graph in their work [7]. Exchange property in resolving set for nanotube discussed in [8]. Double edge resolving set and exchange property for nanosheet discussed by Sikander ali [9]. The study of homogeneous caterpillar graphs with fault-tolerant partition size was carried out in the context of [10]. Hussain et al. delved into the fault-tolerance of lattices of boron nanotubes [11], while Guo discussed certain types of line graph's fault-tolerant resolvability [12]. Chemical graph fault-tolerant partition resolvability was discussed in [13]. Extremal structures and the fault-tolerant resolvability of graphs were subjects of study in [14]. In contrast, the fault-tolerant metric dimension and edge fault-tolerant metric dimension of hollow coronoid structures were addressed in [15]. Finally, the investigation into fault-tolerant resolving sets for certain families of ladder networks was carried out in [16].

In our daily lives, the concept of metric dimension finds a multitude of practical applications that serve as a source of inspiration for researchers and have been widely explored. One noteworthy example is the application of metric dimension in discerning analogous patterns among a diverse range of medications [17]. The versatility of metric dimensions extends to various fields: Combinatorial Optimization: Metric dimension proves valuable in solving combinatorial optimization problems [18], [19]. Robot Navigation: Metric dimension plays a pivotal role in robot navigation [20]. Pharmaceutical Chemistry: The practical implications of metric dimension are evident in the realm of pharmaceutical chemistry [21]. Computer Networks: The metric dimension contributes to understanding and optimizing computer networks [22]. Graph Labeling: The concept of metric dimension is harnessed for canonically labeling graphs [23]. Location Problems: The metric dimension plays a role in addressing location problems, including navigation systems like sonar and Loran [24], [25]. Mastermind Game: Metric dimension is applied to the coding and decoding of the Mastermind game [26]. For a more comprehensive exploration of the physical and chemical attributes linked to the metric dimension, further references delve into these aspects [27], [28], [29], [30], [31].

In the realm of chemistry, the concept of double-resolving sets finds numerous applications, with its exploration spanning across various articles. The investigation into the double-resolving set of distinct chemical structures has been the subject of several studies. The double-resolving set of the Cocktail Party and Jellyfish graphs was examined in the work by [32]. The computation of minimum double-resolving sets of graphs was explored in the research by [33]. Novel resolvability parameter of some well-known graphs and exchange properties with applications discussed by Sikander in [34] and double edge resolving set and exchange property for nanosheet discussed in [35]. Further contributions include the determination of the metric dimension of nanotubes of $VC_{4}C_{7}$ and H-Naphtalenic nanotubes as discussed. The resolving set of silicate stars was examined by [36]. Upper bounds on the metric dimension of cellulose networks were established in the research by [37]. Discussions about the Convex polytopes graph were presented in the work by [38]. The versatility of the concept of metric dimension is demonstrated by its application to solve various intricate problems across different domains. For insights into the resolvability parameters of diverse chemical structures, additional references can be found in sources such as [39], [40], [41].

To explore additional physical and chemical attributes of octagonal grids, further insights can be found in references such as [42], [43], [44]. Exosomes from young healthy human plasma promote functional recovery from intracerebral hemorrhage via counteracting ferroptotic injury [45], [46], [47], [48]. Review of the correlation between Chinese medicine and intestinal microbiota on the efficacy of diabetes mellitus [49], [50], [51], [52].

To facilitate a clearer understanding of the concepts used in this article, here are some fundamental mathematical definitions: Distance: Distance refers to the measure of separation between two points or objects in a space. Its definition is the length of the shortest path in a graph between any two vertices. Resolving Set In a graph, a resolving set is a subset of vertices that, based on their distances from one another, uniquely determines the positions of all other vertices. The lowest cardinality of a resolving set that can definitively identify the locations of every other graph vertice is known as the metric dimension of a graph. These definitions serve as foundational concepts that play a pivotal role in the discussions presented in this article.

Definition 1.1 The shortest path length between vertices $\beta_{1}$ and $\beta_{2}$ in graph G is referred to as the distance, often termed as a geodesic, between these two vertices $\beta_{1},\beta_{2}\in V(G)$. In the context of the simple, undirected graph G, where the sets of vertices and edges are denoted by V(G) and E(G) respectively, the distance between vertices is represented as *η*($\beta_{1},\beta_{2}$).

This article relies on several fundamental Theorems. In the work by [53], the author establishes that dim(G)=1 if and only if *G* is the path graph $P_{n}$. Additionally, in the study conducted by [54], it is demonstrated that $dim(C_{n})=2$ for cycles $C_{n}$ where n≥3.

Lemma 1.1 *Let G be any graph. Then*$\beta (G)<\beta^{\prime }(G)$*where*β(G)*is the metric dimension and*$\beta^{\prime }(G)$*is fault-tolerant metric dimension.*

Lemma 1.2 [55]*For all G,*$\beta^{\prime }(G)\geq 3$*if*$G\neq P_{n}$*, where*$\beta^{\prime }(G)$*is fault-tolerant metric dimension of G.*

## 2 Materials and methods

In this article, we use some abbreviations for names of parameters like *MC* (MC), metric dimension (MD) mixed metric dimension (MMD), resolving set(RS), etc. We compute the resolvability parameter just like the double resolving set, double edge resolving set, and double mixed resolving set of fosmidomycin.

## 3 Results

### 3.1 Metric dimension of anti-malaria drug fosmidomycin

In this section, we compute the resolvability parameters like the double resolving set and exchange property of the molecular graph of fosmidomycin.

#### 3.1.1 Fosmidomycin

**Figure 1:**
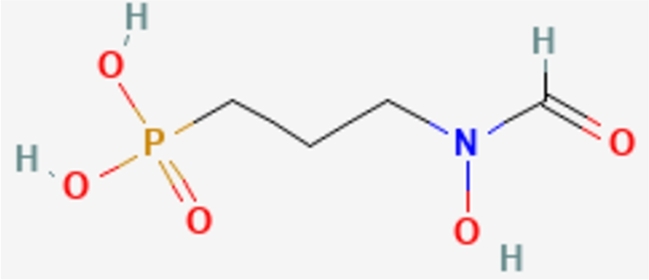
Fosmidomycin Molecular structures.

Fig. 1 is the Molecular structures of fosmidomycin. Here are some properties of fosmidomycin. Fosmidomycin is a medication with various medical uses, primarily known for its role as an antimalarial drug. Here is some information about fosmidomycin: An important part of the bacterial cell wall, 2-keto-3-deoxy-d-manno-octulosonic acid (KDO), has a structural analog in the antibiotic fosmidomycin. Because of its well-known antimalarial qualities, fosmidomycin has been investigated as a possible malaria therapy. These are some salient details regarding fosmidomycin: *Preventive Action:* Fosmidomycin is known to inhibit the enzyme 1-deoxy-D-xylulose-5-phosphate reductoisomerase (DXR), also known as 1-deoxy-D-xylulose-5-phosphate synthase (DXPS). This crucial enzyme plays a vital role in the production of isoprenoids in bacteria as well as in the apicoplast of the malaria parasite Plasmodium falciparum, by utilizing the non-mevalonate pathway. DXR's inhibition by fosmidomycin is significant because it is considered a potential drug target for the treatment of malaria and other diseases caused by bacteria. Fosmidomycin prevents the synthesis of isoprenoids, which is essential for the malaria parasite's survival, by blocking DXR. of isoprenoids, which are necessary for several biological functions, such as the synthesis of membranes and the prenylation of proteins. *Clinical Experiments;* Clinical trials on the antimalarial potential of fosmidomycin, either by itself or in combination with other antimalarial medications, have been conducted. Evaluation of its effectiveness, safety, and tolerance as a possible malaria treatment has been the main focus of research. A*pply to Combination Treatments:* Combining fosmidomycin with other antimalarial medications can increase their effectiveness and lower their chance of developing drug resistance. Studies have been conducted on combinations with medications such as clindamycin. *Hypertrophic Factor:* Fosmidomycin has some inhibitory effects against specific bacteria in addition to its primary target, the malaria parasite. Research on this dual activity's potential for treating bacterial infections has resulted. *Research and Development phase:* Research into fosmidomycin's antimalarial qualities and possible uses in other therapeutic domains is still ongoing.

*Construction of Fosmidomycin Molecular graph*. The color scheme is used for vertices and edge labeling. The blue edges are pendent and the remaining edges are black. The green color vertices are the members of the resolving set, the pink color vertices have a degree of 3 and the remaining vertices are back-colored. The total number of vertices is 12 the total number of edges is 11 and the vertex and edge set is defined as

$$V(FC)=\{a_{i},b_{j};1\leq i\leq 8,1\leq j\leq 4\},E(CQ)=\{a_{i}a_{i+1},a_{i}b_{i};1\leq i\leq 8,1\leq j\leq 4\}.$$

**Figure 2:**
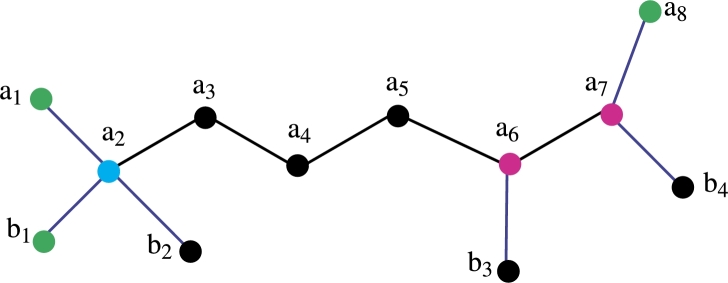
Fosmidomycin Mathematical graph.

In Fig. 2 the green color vertices are the member of the resolving set and the pink color vertices are of degree 3, the sky blue color vertex has degree 4 and the remaining vertices are black. The blue color assigns to the pendant edges and the remaining edges are black.

Theorem 3.1 *If FC is a molecular graph of a fosmidomycin malaria drug. Then exchange property hold in double resolving sets of cardinality* 3*.*

Proof Let $R_{1}$ and $R_{2}$ be two sets of vertices of a fosmidomycin molecular graph. We wish to display the unique representation of both sets to substantiate this assertion. The representation of $R_{1}$ is in Theorem 3.2 and the representation of $R_{2}$ is in Theorem 3.3 is proved. □

Theorem 3.2 *If FC is a molecular graph of a fosmidomycin malaria drug. Then*$R_{1}$*be a resolving set of cardinality* 3*.*

Proof Let $R_{1}$ be a set of vertices of a fosmidomycin molecular graph. We wish to display the unique representation of $R_{1}$ to substantiate this assertion, let's proceed to prove this by demonstrating that the set $R_{1}$ has a unique representation with all the vertices of the fosmidomycin molecular graph *FC*. Let $R_{1}=\{a_{1},b_{1},a_{8}\}$. We now aim to show that $R_{1}$ has a unique representation with all the vertices of *FC*.

The representation of $R_{1}=\{a_{1},b_{1},a_{8}\}$ present in Table 1, Table 2.

**Table 1:** Representation of vertices of Fig. 2.

*r*(.|*R*_1_)	(*a*_1_,*b*_1_,*a*_8_)
*a*_1_	(0, 2, 7)
*a*_2_	(1, 1, 6)
*a*_3_	(2, 2, 5)
*a*_4_	(3, 3, 4)
*a*_5_	(4, 4, 3)
*a*_6_	(5, 5, 2)

**Table 2:** Representation of vertices of Fig. 2.

*r*(.|*R*_1_)	(*a*_1_,*b*_1_,*a*_8_)
*a*_7_	(6, 6, 1)
*a*_8_	(7, 7, 0)
*b*_1_	(2, 0, 7)
*b*_2_	(2, 2, 7)
*b*_3_	(6, 6, 3)
*b*_4_	(7, 7, 2)

After analyzing Table 1 and Table 2, it becomes evident that $R_{1}$ has a unique representation with all the vertices of *FC*. Therefore, $R_{1}$ is considered a *RS* with a cardinality of 3. In this case, the *MC* of $R_{1}$ is the metric dimension, which is 3 for fosmidomycin. Based on the discussion so far, it is clear that the cardinality of the *RS* of *FC* is less than or equal to 3. However, we need to prove that it is not less than 3. The *RS* of cardinality 1 is possible only in the case of a path graph. On the other hand, the *RS* of cardinality 2 is not possible because when we take any set of cardinality two, the representation remains the same for some vertices of fosmidomycin. Hence, we can conclude that $R_{1}$ is the only *RS* with a cardinality of 3. □

Theorem 3.3 *If FC is a molecular graph of a fosmidomycin malaria drug. Then*$R_{2}$*is also a RS of cardinality* 3 *exists.*

Proof Let $R_{2}$ be a set of vertices of a fosmidomycin molecular graph. We wish to display the unique representation of $R_{2}$ to substantiate this assertion, let's proceed to prove this by demonstrating that the set $R_{2}$ has a unique representation with all the vertices of the fosmidomycin molecular graph *FC*. Let $R_{2}=\{a_{1},b_{2},b_{4}\}$. We now aim to show that $R_{2}$ has a unique representation with all the vertices of *FC*.

The representation of $R_{2}=\{a_{1},b_{2},b_{4}\}$ present in Table 3, Table 4.

**Table 3:** Representation of vertices of Fig. 3.

*r*(.|*R*_2_)	(*a*_1_,*b*_2_,*b*_4_)
*a*_1_	(0, 2, 7)
*a*_2_	(1, 1, 6)
*a*_3_	(2, 2, 5)
*a*_4_	(3, 3, 4)
*a*_5_	(4, 4, 3)
*a*_6_	(5, 5, 2)

**Table 4:** Representation of vertices of Fig. 3.

*r*(.|*R*_2_)	(*a*_1_,*b*_2_,*b*_4_)
*a*_7_	(6, 6, 1)
*a*_8_	(7, 7, 2)
*b*_1_	(2, 2, 7)
*b*_2_	(2, 0, 7)
*b*_3_	(6, 6, 3)
*b*_4_	(7, 7, 0)

From Table 3, Table 4 it is clear that the $R_{2}$ has unique Representation with all vertices of *FC* so $R_{2}$ is *RS* of cardinality 3. The *MC* of $R_{2}$ is metric dimension so the metric dimension of fosmidomycin is 3. □

**Figure 3:**
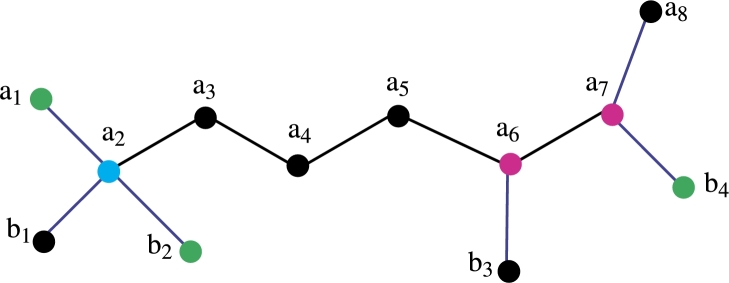
Fosmidomycin Mathematical graph.

**Figure 4:**
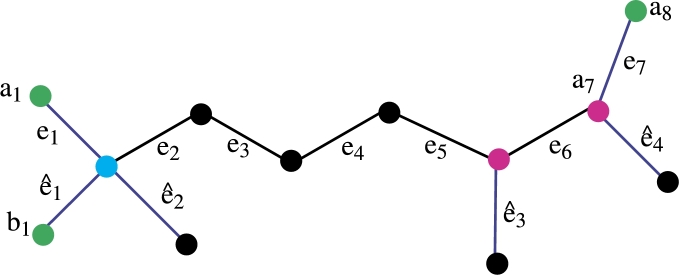
Fosmidomycin Mathematical graph.

**Figure 5:**
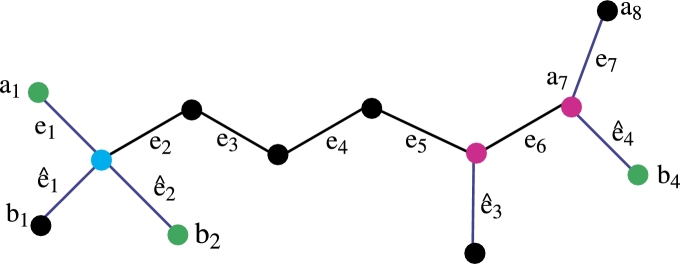
Fosmidomycin Mathematical graph.

**Figure 6:**
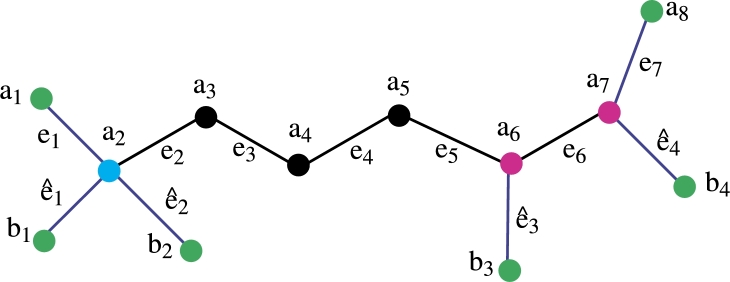
Fosmidomycin Mathematical graph.

**Figure 7:**
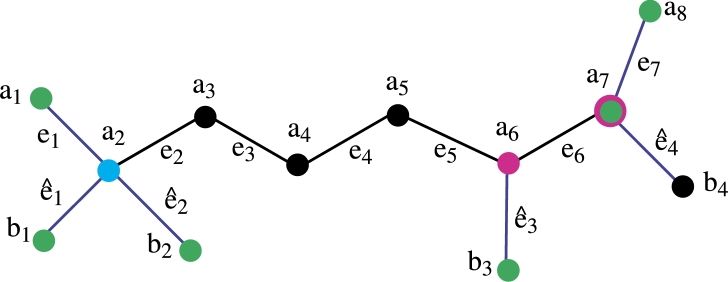
Fosmidomycin Mathematical graph.

#### 3.1.2 Exchange property in *RS*s

If every vertex in a graph G can be distinguished by its distances to the vertices of a subset *W*, then that subset *W* is a *RS*. In a vector space, *RS*s act as bases because they allow for the unique identification of each vertex in the graph concerning the vertices of these sets. Although they share some similarities with bases in a vector space, *RS*s do not necessarily possess the exchange property from linear algebra. The exchange property states that if *S* and *R* are minimum *RS*s for *G* and *r* is an element of *R*, then there exist an element *s* in *S* such that a minimal *RS* for *G* is (S﹨{s})∩{r}. When the exchange property is satisfied, all of graph $G^{\prime }s$ minimal *RS*s have the same size, which makes algorithmic methods for determining $G^{\prime }s$ metric dimension more effective. Therefore, if two minimal *RS*s of different sizes are identified, it is sufficient to prove that the exchange property is not present in the given graph. It is important to note that the opposite is not necessarily true, as minimal *RS*s of different sizes are not always required for the exchange property to be absent. [5] included the following deductions on the exchange property for *RS*s.

Theorem 3.4 *[5]**states that the exchange property holds for RSs in trees.*

Theorem 3.5 *The exchange property is absent from RSs in wheels*$W_{n}$*for*n≥8*[5]**.*

It has been shown recently that the exchange property for solving sets of necklace graphs $Ne_{n}$ does not hold.

Theorem 3.6 *There is no exchange property in the sets of resolving necklace graphs. For*n≥4*[56]**,*$Ne_{n}$

Theorem 3.7 *Quasi-flower snarks for*n≥4*[57]**do not satisfy the exchange property for minimal RSs.*

Theorem 3.8 *In minimal RSs of nanotubes derived from the octagonal grid, the exchange property is held for*h,v≥1*[58]**.*

In the following Theorem, we show that the exchange property holds for sets of resolved fosmidomycin molecular graphs.

Theorem 3.9 *For sets of resolved fosmidomycin molecular graphs, the exchange property holds.*

Proof In fosmidomycin molecular graph we have two *RS*s let $R_{1}=\{a_{1},b_{1},a_{8}\}$ and $R_{2}=\{a_{1},b_{2},b_{4}\}$. According to the definition of exchange property $b_{1}\in R_{1}$ and $b_{2}\in R_{2}$ such that $\left(R_{1}﹨\{b_{1}\}\cap \{b_{2}\}\right)$ is also a resoling set. In these *RS*s, we change two elements. Let $v=\{b_{1},a_{8}\}$, $u=\{b_{2},b_{4}\}$ and $v\in R_{1}$, $u\in R_{2}$ such that $(R_{1}﹨\{v\}\cap \{u\})=R_{2}$. $R_{2}$ is also a *RS* proved in Theorem 3.1. □

### 3.2 Edge metric dimension of fosmidomycin

In this section, we compute the resolvability parameter of the double-edge *RS* and the exchange property of the molecular graph of fosmidomycin.

#### 3.2.1 Fosmidomycin

Theorem 3.10 *If FC is a molecular graph of a fosmidomycin malaria drug. Then exchange property hold in double edge RSs of cardinality* 3*.*

Proof Let $R_{1}$ and $R_{2}$ be two sets of vertices of a fosmidomycin molecular graph. We wish to display the unique representation of both sets with all edges of Fosmidomycin to substantiate this assertion. The representation of $R_{1}$ is in Theorem 3.11 and the representation of $R_{2}$ is in Theorem 3.12 is proved. □

Theorem 3.11 *If FC is a molecular graph of a fosmidomycin malaria drug. Then*$R_{1}$*be an edge resolving a set of cardinality* 3*.*

Proof Let $R_{1}$ be a set of vertices of a fosmidomycin molecular graph. We wish to display the unique representation of $R_{1}$ with all edges of Fosmidomycin to substantiate this assertion, let's proceed to prove this by demonstrating that the set $R_{1}$ has a unique representation with all the edges of the fosmidomycin molecular graph *FC*. Let $R_{1}=\{a_{1},b_{1},a_{8}\}$. We now aim to show that $R_{1}$ has a unique representation with all the edges of *FC*.

The representation of $R_{1}=\{a_{1},b_{1},a_{8}\}$ present in Table 5, Table 6.

**Table 5:** Representation of edges of Fig. 4.

*r*(.|*R*_1_)	(*a*_1_,*b*_1_,*a*_8_)
*e*_1_	(0, 1, 6)
*e*_2_	(1, 1, 5)
*e*_3_	(2, 2, 4)
*e*_4_	(3, 3, 3)
*e*_5_	(4, 4, 2)
*e*_6_	(5, 5, 1)

**Table 6:** Representation of edges of Fig. 4.

*r*(.|*R*_1_)	(*a*_1_,*b*_1_,*a*_8_)
*e*_7_	(6, 6, 0)
${\overset{ˆ}{e}}_{1}$	(1, 0, 6)
${\overset{ˆ}{e}}_{2}$	(1, 1, 6)
${\overset{ˆ}{e}}_{3}$	(5, 5, 2)
${\overset{ˆ}{e}}_{4}$	(6 6, 1)

From Table 5, Table 6 it is clear that the $R_{1}$ has unique Representation with all edges of *FC* so $R_{1}$ is edge *RS* of cardinality 3. The *MC* of $R_{1}$ is the edge metric dimension so the edge metric dimension of fosmidomycin is 3. From all the above discussion it is clear that the metric dimension of *FC* is less or equal to 3. Now we are going to prove that the cardinality of the edge *RS* of *FC* is not less than 3. The cardinality of the edge *RS* is one possible only in the case of a path graph. The cardinality of the edge *RS* is 2 which is not possible because when we take any set of cardinality two the representation remains the same for some edges of fosmidomycin. Hence the cardinality of the edge-*RS* of fosmidomycin is 3. □

Theorem 3.12 *If FC is a molecular graph of a fosmidomycin malaria drug. Then*$R_{2}$*is also be a edge RS of cardinality* 3*.*

Proof Let $R_{2}$ be a set of vertices of a fosmidomycin molecular graph. We wish to display the unique representation of $R_{2}$ with all edges of Fosmidomycin to substantiate this assertion, let's proceed to prove this by demonstrating that the set $R_{2}$ has a unique representation with all the edges of the fosmidomycin molecular graph *FC*. Let $R_{1}=\{a_{1},b_{2},b_{4}\}$. We now aim to show that $R_{2}$ has a unique representation with all the edges of *FC*.

The representation of $R_{2}=\{a_{1},b_{2},b_{4}\}$ present in Table 7, Table 8.

**Table 7:** Representation of edges of Fig. 5.

*r*(.|*R*_2_)	(*a*_1_,*b*_2_,*b*_4_)
*e*_1_	(0, 1, 6)
*e*_2_	(1, 1, 5)
*e*_3_	(2, 2, 4)
*e*_4_	(3, 3, 3)
*e*_5_	(4, 4, 2)
*e*_6_	(5, 5, 1)

**Table 8:** Representation of edges of Fig. 5.

*r*(.|*R*_2_)	(*a*_1_,*b*_2_,*b*_4_)
*e*_7_	(6, 6, 1)
${\overset{ˆ}{e}}_{1}$	(1, 1, 6)
${\overset{ˆ}{e}}_{2}$	(1, 0, 6)
${\overset{ˆ}{e}}_{3}$	(5, 5, 2)
${\overset{ˆ}{e}}_{4}$	(6 6, 0)

From Table 7, Table 8 it is clear that the $R_{2}$ has unique Representation with all edges of *FC* so $R_{2}$ is edge *RS* of cardinality 3. Since $R_{2}$ has *MC* 3, the edge metric dimension of fosmidomycin is 3. It is evident from the entire discussion above that *FC* has a metric dimension of less than or equal to 3. We will now demonstrate that the cardinality of the set that resolves edges in *FC* is more than or equal to 3. Only in the case of a path graph is the cardinality of the edge *RS* one. Since the representation of some edges of fosmidomycin remains the same for any set of cardinality two, the cardinality of the edge *RS*, 2, is not possible. Therefore, 3 is the cardinality of the fosmidomycin edge-*RS*. □

#### 3.2.2 Exchange property in edge *RS*s

We demonstrate that the exchange property is valid for edge-*RS*s of the fosmidomycin molecular graph in the following Theorem.

Theorem 3.13 *The exchange property is valid for edge-RSs of fosmidomycin molecular graphs.*

Proof In the fosmidomycin molecular graph we have two edge *RS*s let $R_{1}=\{a_{1},b_{1},a_{8}\}$ and $R_{2}=\{a_{1},b_{2},b_{4}\}$. According to the definition of exchange property $b_{1}\in R_{1}$ and $b_{2}\in R_{2}$ such that $\left(R_{1}﹨\{b_{1}\}\cap \{b_{2}\}\right)$ is also a edge resoling set. In these *RS*s, we change two elements. Let $v=\{b_{1},a_{8}\}$, $u=\{b_{2},b_{4}\}$ and $v\in R_{1}$, $u\in R_{2}$ such that $(R_{1}﹨\{v\}\cap \{u\})=R_{2}$. $R_{2}$ is also a edge *RS* proved in Theorem 3.1. □

### 3.3 Mixed metric dimension of fosmidomycin

In this section, we compute the resolvability parameter of the double mixed *RS* and the exchange property of the molecular graph of fosmidomycin.

#### 3.3.1 Fosmidomycin

Theorem 3.14 *If FC is a molecular graph of a fosmidomycin malaria drug. Then exchange property hold in double mixed RSs of cardinality* 6*.*

Proof Let $R_{1}$ and $R_{2}$ be two sets of vertices of a fosmidomycin molecular graph. We wish to display the unique representation of both sets to substantiate this assertion. The representation of $R_{1}$ is in Theorem 3.15 and the representation of $R_{2}$ is in Theorem 3.16 is proved. □

Theorem 3.15 *If FC is a molecular graph of a fosmidomycin malaria drug. Then*$R_{1}$*be a mixed RS of cardinality* 6*.*

Proof Let $R_{1}$ be a set of vertices of a fosmidomycin molecular graph. We wish to display the unique representation of $R_{1}$ with all vertices and edges of *FC* to substantiate this assertion, let's proceed to prove this by demonstrating that the set $R_{1}$ has a unique representation with all the vertices and edges of the fosmidomycin molecular graph *FC*. Let $R_{1}=\{a_{1},b_{1},a_{8}\}$. We now aim to show that $R_{1}$ has a unique representation with all the vertices of *FC*.

The representation of $R_{1}=\{a_{1},b_{1},b_{2},b_{3},b_{4},a_{8}\}$ present in Table 9, Table 10.

**Table 9:** Representation of vertices of Fig. 6.

*r*(.|*R*_1_)	(*a*_1_,*b*_1_,*b*_2_,*b*_3_,*b*_4_,*a*_8_)
*a*_1_	(0, 2, 2, 6, 7, 7)
*a*_2_	(1, 1, 1, 5, 6, 6)
*a*_3_	(2, 2, 2, 4, 5, 5)
*a*_4_	(3, 3, 3, 3, 4, 4)
*a*_5_	(4, 4, 4, 2, 3, 3)
*a*_6_	(5, 5, 5, 1, 2, 2)
*a*_7_	(6, 6, 6, 2, 1, 1)
*a*_8_	(7, 7, 7, 3, 2, 0)
*b*_1_	(2, 0, 2, 6, 7, 7)
*b*_2_	(2, 2, 0, 6, 7, 7)
*b*_3_	(6, 6, 6, 0, 3, 3)
*b*_4_	(7, 7, 7, 3, 0, 2)

**Table 10:** Representation of edges of Fig. 7.

*r*(.|*R*_1_)	(*a*_1_,*b*_1_,*b*_2_,*b*_3_,*b*_4_,*a*_8_)
*e*_1_	(0, 1, 1, 5, 6, 6)
*e*_2_	(1, 1, 1, 4, 5, 6)
*e*_3_	(2, 2, 2, 3, 4, 4)
*e*_4_	(3, 3, 3, 2, 3, 3)
*e*_5_	(4, 4, 4, 1, 2, 2)
*e*_6_	(5, 5, 5, 1, 1, 1)
*e*_7_	(6, 6, 6, 2, 1, 0)
${\overset{ˆ}{e}}_{1}$	(1, 0, 1, 5, 6, 6)
${\overset{ˆ}{e}}_{2}$	(1, 1, 0, 5, 6, 6)
${\overset{ˆ}{e}}_{3}$	(5, 5, 5, 0, 2, 2)
${\overset{ˆ}{e}}_{4}$	(6, 6, 6, 2, 0, 1)

From Table 9, Table 10 it is clear that the R1 has unique Representation with all vertices and edges of *FC* so $R_{1}$ is mixed *RS* of cardinality 6. The *MC* of $R_{1}$ is a *MMD* so the *MMD* of fosmidomycin is 6. From all the above discussion it is clear that the cardinality of a mixed *RS* of *FC* is less or equal to 3. Now we are going to prove that it is not less than 6. The mixed *RS* of cardinality 1 is possible only in the case of a path graph. The mixed *RS* of cardinality 2 is not possible because when we take any set of cardinality two the representation remains the same for some vertices of fosmidomycin. Hence $R_{1}$ is the mixed *RS* of cardinality 3. □

Theorem 3.16 *If FC is a molecular graph of a fosmidomycin malaria drug. Then*$R_{2}$*is also a mixed RS of cardinality* 6*.*

Proof Let $R_{2}$ be a set of vertices of a fosmidomycin molecular graph. We wish to display the unique representation of $R_{2}$ with all vertices and edges of *FC* to substantiate this assertion, let's proceed to prove this by demonstrating that the set $R_{2}$ has a unique representation with all the vertices and edges of the fosmidomycin molecular graph *FC*. Let $R_{2}=\{a_{1},b_{2},b_{4}\}$. We now aim to show that $R_{2}$ has a unique representation with all the vertices and edges of *FC*.

The representation of $R_{1}=\{a_{1},b_{1},b_{2},b_{3},a_{7},a_{8}\}$ present in Table 11, Table 12.

**Table 11:** Representation of vertices of Fig. 7.

*r*(.|*R*_1_)	(*a*_1_,*b*_1_,*b*_2_,*b*_3_,*a*_7_,*a*_8_)
*a*_1_	(0, 2, 2, 6, 6, 7)
*a*_2_	(1, 1, 1, 5, 5, 6)
*a*_3_	(2, 2, 2, 4, 4, 5)
*a*_4_	(3, 3, 3, 3, 3, 4)
*a*_5_	(4, 4, 4, 2, 2, 3)
*a*_6_	(5, 5, 5, 1, 1, 2)
*a*_7_	(6, 6, 6, 2, 0, 1)
*a*_8_	(7, 7, 7, 3, 1, 0)
*b*_1_	(2, 0, 2, 6, 6, 7)
*b*_2_	(2, 2, 0, 6, 6, 7)
*b*_3_	(6, 6, 6, 0, 2, 3)
*b*_4_	(7, 7, 7, 3, 1, 2)

**Table 12:** Representation of edges of Fig. 7.

*r*(.|*R*_1_)	(*a*_1_,*b*_1_,*b*_2_,*b*_3_,*a*_7_,*a*_8_)
*e*_1_	(0, 1, 1, 5, 5, 6)
*e*_2_	(1, 1, 1, 4, 4, 6)
*e*_3_	(2, 2, 2, 3, 3, 4)
*e*_4_	(3, 3, 3, 2, 2, 3)
*e*_5_	(4, 4, 4, 1, 1, 2)
*e*_6_	(5, 5, 5, 1, 0, 1)
*e*_7_	(6, 6, 6, 2, 0, 0)
${\overset{ˆ}{e}}_{1}$	(1, 0, 1, 5, 5, 6)
${\overset{ˆ}{e}}_{2}$	(1, 1, 0, 5, 5, 6)
${\overset{ˆ}{e}}_{3}$	(5, 5, 5, 0, 1, 2)
${\overset{ˆ}{e}}_{4}$	(6, 6, 6, 2, 0, 1)

From Table 11, Table 12 it is clear that the $R_{2}$ has unique Representation with all vertices and edges of *FC* so $R_{2}$ is mixed *RS* of cardinality 6. The *MC* of $R_{2}$ is *MMD* so the *MMD* of fosmidomycin is 6. □

#### 3.3.2 Exchange property in mixed *RS*s

We demonstrate that the exchange property is valid for mixed *RS*s of the fosmidomycin molecular graph in the following Theorem.

Theorem 3.17 *The exchange property is valid for mixed RSs of fosmidomycin molecular graphs.*

Proof In the fosmidomycin molecular graph, we have two mixed *RS*s let $R_{1}=\{a_{1},b_{1},b_{2},b_{3},b_{4},a_{8}\}$ and $R_{2}=\{a_{1},b_{1},b_{2},b_{3},a_{7},a_{8}\}$. According to the definition of exchange property $b_{4}\in R_{1}$ and $a_{7}\in R_{2}$ such that $\left(R_{1}﹨\{b_{4}\}\cap \{a_{7}\}\right)$ is also a mixed resoling set. In these mixed *RS*s we change elements *v* and *u*. Let $v=\{b_{4}\}$, $u=\{a_{7}\}$ and $v\in R_{1}$, $u\in R_{2}$ such that $(R_{1}﹨\{v\}\cap \{u\})=R_{2}$. $R_{2}$ is also a mixed *RS* proved in Theorem 3.16. □

## 4 Conclusions

In this article, we talk about fosmidomycin anti-malaria drug. Based on the graph's distance, we determine some resolvability parameters double *RS* and exchange property, double edge *RS* and exchange property, and double mixed *RS* and exchange property for fosmidomycin. The double *RS* and double edge *RS* have cardinality 3, but double mixed *RS* for fosmidomycin have a cardinality 6. The exchange property in this article used for $R_{1}$ and $R_{2}$ then both remain show resolvability.

## CRediT authorship contribution statement

**Rashad Ismail:** Writing – review & editing, Writing – original draft, Formal analysis, Data curation, Conceptualization. **Sikander Ali:** Writing – review & editing, Writing – original draft, Methodology, Investigation, Funding acquisition. **Muhammad Azeem:** Writing – review & editing, Writing – original draft, Visualization, Validation, Supervision. **Manzoor Ahmad Zahid:** Writing – review & editing, Writing – original draft, Software, Resources, Project administration.

## Declaration of Competing Interest

The authors declare that they have no known competing financial interests or personal relationships that could have appeared to influence the work reported in this paper.

## Data Availability

No data is available.
